# Fresh fruit consumption and all-cause and cause-specific mortality: findings from the China Kadoorie Biobank

**DOI:** 10.1093/ije/dyx042

**Published:** 2017-04-24

**Authors:** Huaidong Du, Liming Li, Derrick Bennett, Ling Yang, Yu Guo, Timothy J Key, Zheng Bian, Yiping Chen, Robin G Walters, Iona Y Millwood, Junshi Chen, Junzheng Wang, Xue Zhou, Le Fang, Yijun Li, Xianzhi Li, Rory Collins, Richard Peto, Zhengming Chen, on behalf of the China Kadoorie Biobank study

**Affiliations:** 1 Medical Research Council Population Health Research Unit, Nuffield Department of Population Health, University of Oxford, Oxford, UK; 2 Clinical Trial Service Unit and Epidemiological Studies Unit, Nuffield Department of Population Health, University of Oxford, Oxford, UK; 3 Department of Epidemiology and Biostatistics, School of Public Health, Peking University Health Science Center, Beijing, China; 4 Chinese Academy of Medical Sciences, Beijing, China; 5 Cancer Epidemiology Unit, Nuffield Department of Population Health, University of Oxford, Oxford, UK; 6 China National Center For Food Safety Risk Assessment, Beijing, China; 7 Licang CDC, Qingdao, Shandong Province, China; 8 Heilongjiang CDC, Harbin, Heilongjiang Province, China; 9 Zhejiang CDC, Hangzhou, Zhejiang Province, China; 10 Meilan CDC, Haikou, Hainan Province, China; 11 Liuyang CDC, Liuyang, Hunan Province, China

**Keywords:** Fruit, mortality, cohort studies, prospective studies, China

## Abstract

**Background:**

Higher fruit consumption is associated with lower risk of cardiovascular disease (CVD). Substantial uncertainties remain, however, about the associations of fruit consumption with all-cause mortality and mortality from subtypes of CVD and major non-vascular diseases, especially in China.

**Methods:**

In 2004–08, the nationwide China Kadoorie Biobank Study recruited > 0.5 million adults aged 30–79 years from 10 diverse localities in China. Fresh fruit consumption was estimated using an interviewer-administered electronic questionnaire, and mortality data were collected from death registries. Among the 462 342 participants who were free of major chronic diseases at baseline, 17 894 deaths were recorded during ∼ 7 years of follow-up. Cox regression yielded adjusted rate ratios (RRs) for all-cause and cause-specific mortality associated with fruit consumption.

**Results:**

At baseline, 28% of participants reported consuming fruit ≥ 4 days/week (regular consumers) and 6% reported never/rarely consuming fruit (non-consumers). Compared with non-consumers, regular consumers had 27% [RR = 0.73, 95% confidence interval (CI) 0.70–0.76] lower all-cause mortality, 34% lower CVD mortality (*n* = 6166; RR = 0.66, 0.61–0.71), 17% lower cancer mortality (*n* = 6796; RR = 0.83, 0.78–0.89) and 42% lower mortality from chronic obstructive pulmonary disease (COPD) (*n* = 1119; RR = 0.58, 0.47–0.71). For each of the above, there was an approximately log-linear dose-response relationship with amount consumed. For mortality from site-specific cancers, fruit consumption was inversely associated with digestive tract cancer (*n* = 2265; RR = 0.72, 0.64–0.81), particularly oesophageal cancer (*n* = 801; RR = 0.65, 0.50–0.83), but not with cancer of lung or liver.

**Conclusions:**

Among Chinese adults, higher fresh fruit consumption was associated with significantly lower mortality from several major vascular and non-vascular diseases. Given the current low population level of fruit consumption, substantial health benefits could be gained from increased fruit consumption in China.

## Introduction

In China, adult mortality has been decreasing in the past several decades, driven mainly by reductions in several major chronic non-communicable diseases such as chronic obstructive pulmonary disease (COPD), haemorrhagic stroke and certain cancers (e.g. oesophageal and stomach cancer).^1^ These decreasing mortality trends are due to many social, dietary, occupational and health care changes, and may well continue (except perhaps in male smokers^2^), if there are further favourable changes in diet and other main causes of chronic diseases at the population level.^1^

Despite recent improvements in nutrition, fruit consumption remains much lower in China than in most Western populations.^3^ Low fruit consumption has been identified as a major risk factor for mortality in many populations.^4^ A recent report estimated that a poor diet, characterized mainly by low fruit and whole grains and high sodium, accounted for about a third of all premature deaths in China in 2010.^1^ These estimates were, however, mainly based on extrapolation of risk estimates from Western populations or were derived indirectly from the associations with major risk factors for chronic diseases (e.g. blood pressure). In Western populations, although the association of fruit consumption with cardiovascular disease (CVD) mortality is relatively well established,^5^ substantial uncertainty remains about its associations with certain CVD subtypes (e.g. haemorrhagic stroke) and with other common chronic diseases (e.g. COPD and cancer). Appropriate understanding of the relationships between fruit consumption and cause-specific mortality is particularly relevant for China, where lifestyle (including diet) and the main disease patterns differ appreciably from those in the West; for example, in China there are relatively high rates of COPD, even among never smokers, and of stroke, particular haemorrhagic stroke.

Previous studies in Western populations have tended to combine total fruit (including fresh and processed fruit) and vegetables. However, fruit and vegetables are often consumed in different settings and ways, particularly in China, with fruit consumed almost exclusively raw as snacks and vegetables usually cooked (with added cooking oil and salt) as part of a meal. In China, although the population level of fruit consumption is relatively low, fresh vegetables are typically consumed daily by nearly all adults.^3^ It is therefore important to understand whether fruit consumption is associated with major health outcomes in this population. So far, very limited prospective evidence addressing this question is available. In the China Kadoorie Biobank (CKB), a large nationwide prospective cohort study, higher fresh fruit consumption has recently been associated with lower risk of CVD incidence and mortality.^6^ We present here relevant findings on the associations of fresh fruit consumption with all-cause and cause-specific mortality in CKB, including mortality from different subtypes of CVDs as well as other major diseases, such as cancers and COPD.

## Methods

### Baseline survey

CKB design, survey methods and participant characteristics have been reported previously.^7^ Briefly, the study was conducted in 10 geographically diverse regions (five urban and five rural) in China, chosen to cover a wide range of risk exposures and disease patterns, all with good quality death registries. Between June 2004 and July 2008, all residents aged 35–74 years from 100–150 administrative units (rural villages or urban residential committees) in each area were invited to attend the study assessment clinics, and about one in three responded and were enrolled. A total of 512 891 participants were recruited, including 12 668 just outside the pre-specified 35–74 years age range, making the age range at enrolment 30–79 years. All participants provided written informed consent. Prior local, national and international ethics approvals were obtained.^7^^,^^8^

At local assessment clinics, trained health workers administered a laptop-based questionnaire on socio-demographic status, lifestyle factors (including smoking, drinking, diet and physical activity) and medical history; measured anthropometrics and blood pressure; and took blood for measurements of blood glucose and long-term storage. Dietary data covered 12 major food groups including staple foods, meat, dairy products, fresh and preserved vegetables and fresh fruit, with five categories of intake frequency (daily, 4–6 days/week, 1–3 days/week, monthly or never/rarely). The Spearman coefficient of reproducibility for fresh fruit consumption was 0.55.^6^

### Resurveys

Following completion of the baseline survey, two resurveys of randomly selected subsamples of surviving participants were undertaken using similar procedures. The first (*n* = 19 788) took place during July–October 2008 and the second during August 2013–September 2014. In the second resurvey (*n* = 25 011), the consumption amount of each food group was also collected, allowing us to estimate average consumption of each baseline frequency category (eTable 1, available as Supplementary data at *IJE* online).^6^

### Mortality follow-up

Cause-specific mortality was monitored periodically through China’s Center for Disease Control (CDC) Disease Surveillance Points system,^9^ checked annually against local residential, medical and health insurance records and confirmed through street committee or village administrators. For the few deaths (∼ 5%) without recent medical attention, standardized verbal autopsy was used to determine probable causes from symptoms or signs described by informants (usually family members). Deaths were International Classification of Diseases (ICD) -10 coded by trained staff, blinded to baseline information (eTable 2). By 1 January 2014, 25 488 (5.0%) participants had died and 2411 (0.5%) were lost to follow-up (censored in analyses).

### Statistical analysis

The present study excluded participants with a baseline history of major chronic diseases, including ischaemic heart disease (IHD, *n* = 15 472), stroke (*n* = 8884), cancer (*n* = 2577), COPD (*n* = 13 289) or diabetes (*n* = 16 162), leaving 462 342 participants for analyses (eFigure 1, available as Supplementary data at *IJE* online).

Multiple linear (for continuous outcomes) or logistic regression (for binary outcomes) was used to compare age, sex and study area adjusted means (standard deviations) or percentages of various baseline characteristics by frequency levels of fresh fruit consumption at baseline, namely never/rarely, monthly, weekly (1–3 days/week) and regularly (≥  4 days/week).

Cox regression, stratified by age-at-risk (5-year intervals), sex and study area (10 regions), was used to calculate mortality rate ratios (RRs) and 95% confidence intervals (CIs) by fruit consumption, adjusting for education (no formal education, primary school, middle or high school, or university), annual household income (< 10 000, 10 000–19 999, 20 000–34 999 or ≥ 35 000 yuan), smoking (never, former, occasional or current regular smokers), alcohol drinking (never, former, occasional or current regular drinkers), physical activity (continuous), consumption of meat (less than weekly, weekly, regularly), dairy products (never/rarely, monthly, at least weekly) and preserved vegetables (in the original five categories), survey season (spring, summer, autumn or winter) and baseline body mass index (BMI, continuous). The floating absolute risk method was used to estimate variance of log risk (thus the CIs of the RRs) in all categories, including the reference category, of fruit consumption. This method allows valid comparisons to be made between any two exposure groups for polychotomous risk factors, rather than just with an arbitrarily chosen reference group.^10^^,^^11^ The proportional hazard assumption was fulfilled, as similar RRs were observed in the first and second half of follow-up.^12^

To account for regression dilution bias^13^ we used resurvey data to estimate the mean usual consumption for each baseline consumption category (eTable 1). The RR for each consumption category was plotted against the level of mean usual consumption and the effect size per daily portion (100 g/day) was estimated. Stratified analyses by potential effect modifiers were performed to examine the RRs related to one daily portion of fruit across different population subgroups. Significance of a trend or heterogeneity test was evaluated using a chi-square test. Sensitivity analyses adjusted for additional dietary variables and excluded the first two years of follow-up and those with prevalent diseases or poor self-rated health at baseline. To assess the specificity of the association, we further analysed the association of fruit consumption with mortality from traffic accidents, which can be considered as a negative control. Analyses used SAS (version 9.2), and graphs were plotted in R 3.0.2.

## Results

Of the 462 342 participants included, the mean (SD) baseline age was 51 (10.5) years; 59% were women and 43% were from urban areas. Overall, 6% reported never/rarely consuming fresh fruit (non-consumers) and 28% reported a regular consumption (i.e. ≥ 4 days/week), with a higher proportion of regular consumers in women (31% vs 23% in men) and in urban areas (47% vs 13% in rural areas). People with more frequent fruit consumption were younger, better educated and less likely to smoke, drink or have poor self-rated health. Compared with non-consumers, regular consumers had 0.6 kg/m^2^ higher BMI but 3.1 mmHg lower mean systolic blood pressure (SBP) (4.1 mmHg after adjusting for BMI) (Table 1).

**Table 1:** Baseline characteristics of participants by frequency of fresh fruit consumption

	Frequency of fresh fruit consumption				
	Never/rarely (*n* = 27534)	Monthly (*n* = 159176)	Weekly (*n* = 147155)	Regularly (*n* = 128477)	Overall (*n* = 462342)
Age, years (SD)	53.7 (10.5)	51.9 (11.0)	50.0 (10.4)	49.2 (11.3)	50.7 (10.5)
Women, %	46.8	56.2	57.0	68.3	59.3
Urban residence, %	29.0	21.4	41.5	72.9	42.6
Education > 9 years, %	11.1	13.3	20.2	33.0	20.9
Household income ≥ 35 000 yuan/year, %	11.6	12.8	16.8	27.2	18.0
Smoking, % men					
Ex-regular	8.5	10.2	12.0	14.2	11.6
Current regular	75.1	68.0	61.0	53.5	63.0
Smoking, % women					
Ex-regular	0.7	0.7	0.7	0.7	0.7
Current regular	3.8	2.8	2.2	1.4	2.2
Alcohol drinking, % men					
Ex-regular	3.1	2.9	2.9	3.4	3.0
Current regular	45.3	37.2	32.9	28.4	34.4
Alcohol drinking, % women					
Ex-regular	0.5	0.3	0.4	0.5	0.4
Current regular	2.8	2.0	2.0	2.3	2.1
Regular consumption of selected food stuff,^a^ %					
Fresh vegetables	92.6	91.1	95.9	98.0	94.6
Preserved vegetables	28.3	21.5	21.5	23.3	22.4
Meat	38.3	39.0	47.2	59.0	47.1
Dairy products	5.6	5.2	8.3	21.5	10.7
Egg	17.6	17.8	21.5	35.4	23.8
Fish	4.9	6.9	8.2	13.5	9.0
Poultry	18.0	17.1	30.1	41.7	28.1
Soybean	7.5	7.3	8.0	14.3	9.5
Physical activity, MET-h/day (SD)	22.0 (12.2)	22.4 (12.8)	21.9 (12.1)	21.1 (13.2)	21.8 (13.9)
Body mass index, kg/m^2^ (SD)	23.2 (3.3)	23.4 (3.4)	23.7 (3.2)	23.8 (3.5)	23.6 (3.3)
Systolic blood pressure, mmHg (SD)	132.1 (19.7)	130.8 (20.6)	130.5 (19.5)	129.0 (21.2)	130.3 (20.9)
Random blood glucose,^b^ mmol/l (SD)	5.97 (1.88)	5.91 (1.97)	5.86 (1.86)	5.81 (2.03)	5.87 (1.89)
Prevalent diseases,^c^ %	15.9	14.5	14.8	16.4	15.2
Self-rated poor health, %	14.9	9.5	7.5	6.7	8.4

Values are either percentage or mean ± standard deviation (SD) and were adjusted for age, sex, and study area where appropriate.

MET, metabolic equivalent.

^a^At least 4 days/week consumption, except for fresh vegetables (daily) and poultry (at least 1 day/week).

^b^Excluding 7594 participants with missing value.

^c^Including those with a history of self-reported physician-diagnosed rheumatic heart disease, rheumatoid arthritis, tuberculosis, asthma, cirrhosis, chronic hepatitis, peptic ulcer, gall/bladder stone, kidney disease, psychiatric disorder or neurasthenia.

During 3.3 million person-years of follow-up, 17 894 deaths were recorded at ages 35–79 years, including 3646 from stroke, 2038 from IHD, 6796 from cancer and 1119 from COPD. Of all cancer deaths, lung cancer was the leading cause followed by liver, stomach, oesophageal and colorectal cancers (eTable 2, available as Supplementary data at *IJE* online).

**Figure 1:**
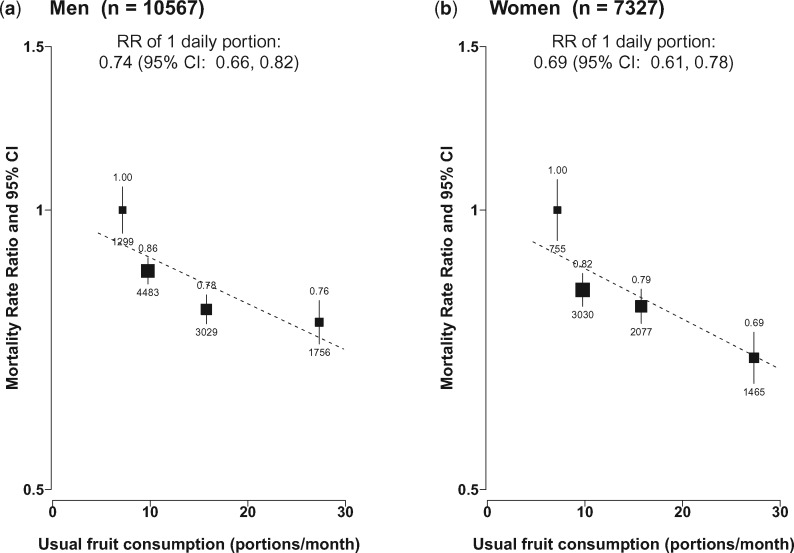
Sex-specific RRs for all-cause mortality by levels of fresh fruit consumption. Analyses were stratified by age-at-risk and study area and adjusted for education, income, alcohol consumption status, smoking, physical activity, intakes of dairy products, meat and preserved vegetables, survey season and BMI. Black boxes represent the point estimates of RRs, with the size inversely proportional to the variance of the log RRs, and the vertical lines represent the 95% confidence intervals (CIs). Values above the vertical lines are RR point estimates and values below them are numbers of deaths.

Fruit consumption was inversely associated with all-cause mortality, in a clear dose-response relationship manner with frequency and amount consumed. Compared with non-consumers, the adjusted RRs were 0.85 (95% CI 0.83–0.87), 0.79 (0.76–0.81) and 0.73 (0.70–0.76), respectively, for monthly, weekly and regular consumers (*P* for trend < 0.0001, eTable 3). After adjusting for regression dilution bias, the association with usual amount of fruit consumed was approximately log-linear, with each daily portion (∼ 100 g) of usual intake associated with 28% (RR = 0.72, 0.66–0.78) lower all-cause mortality. The strength of the association was similar in men and women (Figure 1) and in both urban and rural areas (eFigure 2, available as Supplementary data at *IJE* online). If these associations are largely causal, then 9.4% of all deaths at ages 35–79 years could be attributed to low fruit intake (i.e. non-regular consumption).

**Figure 2:**
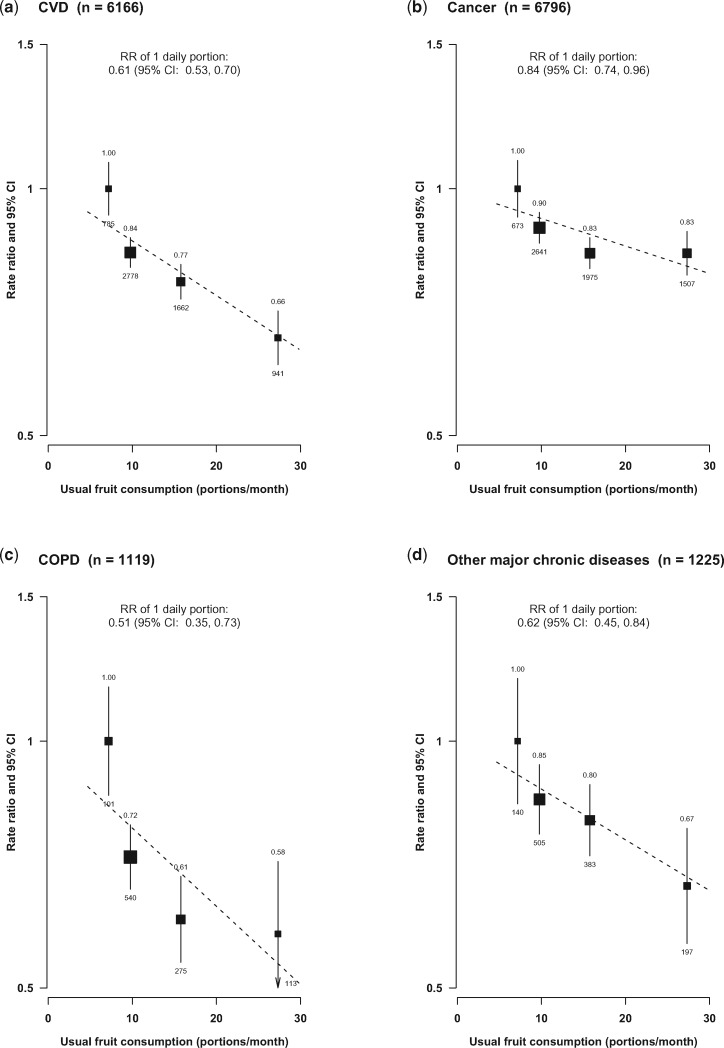
Adjusted RRs for major causes of mortality by levels of fresh fruit consumption. Conventions as in Figure 1. Analyses were stratified by sex, age-at-risk and study area, and adjusted for the same variables as in Figure 1.

Regular fruit consumption was associated with significantly reduced mortality from CVD (0.66, 0.61–0.71), cancer (0.83, 0.78–0.89), COPD (0.58, 0.47–0.71) and an aggregate of several other major chronic diseases (0.67, 0.57–0.78) including mainly diabetes, chronic kidney disease and liver cirrhosis (Figure 2) (eTable 3, available as Supplementary data at *IJE* online). For each there was an approximately log-linear, dose-response relationship with amount consumed, with RRs per one daily portion of usual fruit consumption being 0.61 (0.53–0.70), 0.84 (0.74–0.96), 0.51 (0.35–0.73) and 0.62 (0.45–0.84), respectively, for these four main disease categories (all *P* for trend < 0.01). When comparing those who had low consumption (i.e. < 4 days/week) with regular consumers (i.e. ≥ 4 days/week), the adjusted RR was 1.13 for all-cause mortality and 1.24 for CVD mortality (eTable 4, available as Supplementary data at *IJE* online).

**Figure 3:**
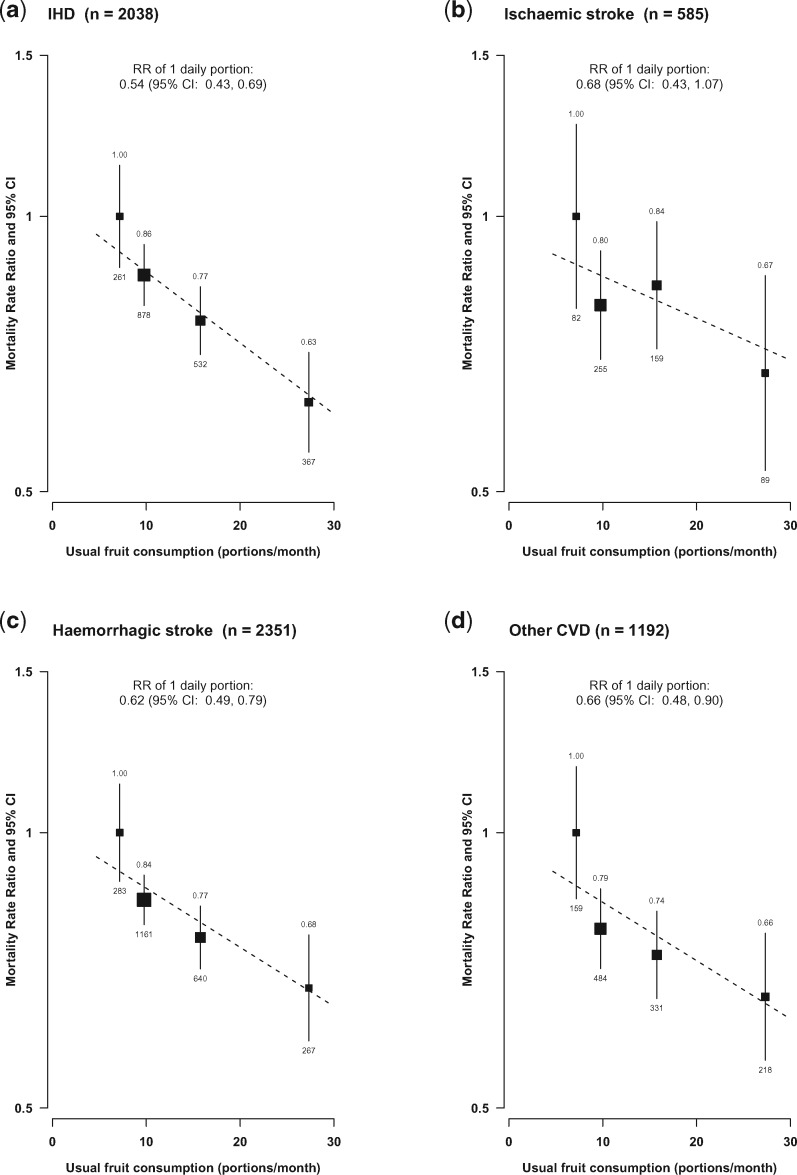
Adjusted RRs for mortality from CVD subtypes by levels of fresh fruit consumption. Conventions as in Figure 2.

For CVD, regular fruit consumption was associated with about one-third lower mortality from IHD, ischaemic stroke, haemorrhagic stroke and other CVD (Figure 3), with adjusted RRs per daily portion of usual fruit consumption being 0.54 (0.43–0.69), 0.68 (0.43–1.07), 0.62 (0.49–0.79) and 0.66 (0.48–0.90), respectively, although the RR for ischaemic stroke had a wide confidence interval due to the relatively small number of deaths (*n* = 585).

**Figure 4:**
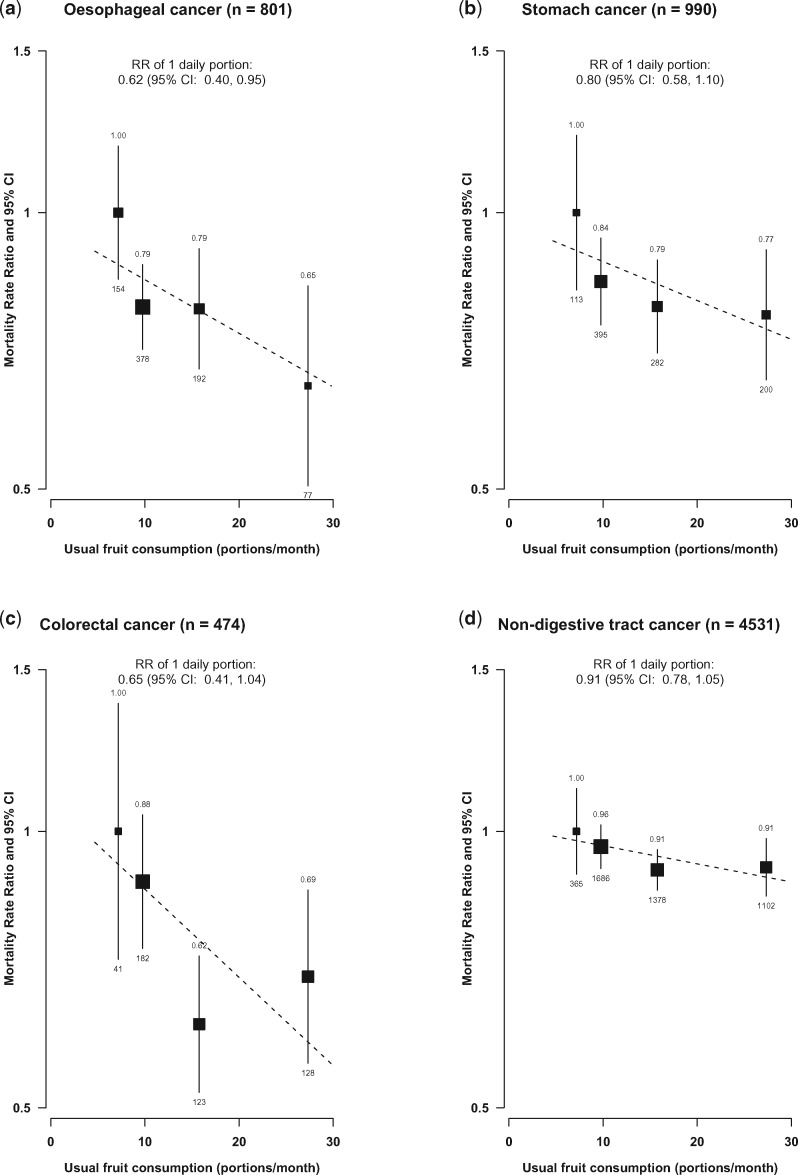
Adjusted RRs for mortality from cancer subtypes by levels of fresh fruit consumption. Conventions as in Figure 2.

For cancer, the inverse association with fruit consumption was mainly driven by digestive tract cancer including cancers of the oesophagus, stomach and colorectum, with each daily portion of usual fruit consumption associated with 28% lower risk (0.72, 0.57–0.90). Of the different types of digestive tract cancer, the inverse association with oesophageal cancer was the strongest [RR per one daily portion fruit = 0.62 (0.40–0.95)] (Figure 4) (eTable 3, available as Supplementary data at *IJE* online). By contrast, fruit consumption was not associated with liver cancer or lung cancer. For an aggregate of all other cancers, regular consumers had a 21% lower risk (RR = 0.79, 0.70–0.88).

For CVD mortality, the strength of the association was highly consistent across different population subgroups (eFigure 3, available as Supplementary data at *IJE* online). For COPD, however, the association was much stronger in women than in men (RR 0.25 vs 0.81, *P* for heterogeneity = 0.002) (eFigure 4, available as Supplementary data at *IJE* online) but this gender difference disappeared after including non-fatal COPD cases (eTable 5, available as Supplementary data at *IJE* online). For cancer, regular fruit intake was associated with increased risk in ex-regular alcohol drinkers (RR = 2.08, 1.20–3.59, *P* for heterogeneity = 0.004) (eFigure 5, available as Supplementary data at *IJE* online), which persisted even after including non-fatal cancer cases (eTable 4).

Additional adjustment for other dietary variables did not alter the results (eTable 6, available as Supplementary data at *IJE* online). For CVD and cancer mortality, exclusion of the first two years of follow-up or participants with other prevalent diseases (such as rheumatic heart disease, chronic hepatitis or kidney disease) or with self-rated poor health status at baseline did not materially alter most of the observed associations either (eTable 7, available as Supplementary data at *IJE* online). For COPD, however, after applying the above three criteria, more than half of the deaths were excluded and its association with fruit consumption was attenuated to null. However, including non-fatal incident cases of COPD in the analysis strengthened the association again (eTable 7). There was no association observed between fruit consumption and mortality from traffic accidents (eTable 3).

## Discussion

This large prospective study in China showed that fresh fruit consumption is associated with significantly lower overall mortality and mortality from a range of cardiovascular and non-cardiovascular diseases. In our study population with almost universal daily consumption of fresh vegetables, those reported consuming fresh fruit regularly (less than one-third of participants) had a 27% lower all-cause mortality than non-consumers, driven mainly by reduced mortality from CVD, COPD, digestive tract cancer and an aggregate of several other major chronic diseases. For each mortality outcome, there was a clear dose-response relationship with amount consumed and the associations appeared to be similar in men and women and in other subgroups of participants.

Several large prospective studies of mainly Western populations have found relatively consistently that higher fruit consumption is associated with lower all-cause and CVD mortality.^5^^,^^6^^,^^14–17^ However, there are large quantitative differences in the strength of the associations observed in different studies and between our results and previous findings. Unlike the present study, previous studies (many of which were small in size) tended to focus on total fruit (i.e. combining fresh and processed fruit) rather than just fresh fruit intake and did not correct for regression dilution bias. In a recent meta-analysis involving ∼ 56 000 deaths from 16 prospective cohort studies, total fruit intake was only weakly associated with all-cause and CVD mortality, with RRs of 0.94 (0.90–0.98) and 0.95 (0.91–1.00), respectively, per 80 g/day of fruit intake.^5^ For stroke, previous Western studies included a limited number of haemorrhagic stroke cases.^18–20^ In a meta-analysis of 20 cohort studies with ∼ 1800 incident haemorrhagic stroke cases and nearly 10 000 ischaemic stroke cases, total fruit and vegetable consumption was inversely and similarly associated with risks of both subtypes of stroke.^21^ Our study provides strong new evidence that regular fruit consumption is associated with significantly lower mortality from stroke, especially haemorrhagic stroke which has high rates in Chinese adults, similar in magnitude to that for IHD.

For cancer, the findings from previous studies are less convincing. In the above-mentioned meta-analysis^5^ that included seven studies with cancer mortality data, none of the three largest studies (i.e. each with > 1000 cancer deaths) observed an association,^17^^,^^22^^,^^23^ including the study conducted in Shanghai, China.^17^ However, in another meta-analysis of ∼ 1.8 million participants with > 15 000 incident lung cancer cases, each daily portion (∼ 80 g) of fruit was associated with 5% lower risk of lung cancer incidence.^24^ Some studies suggest that associations between fruit consumption and cancer were mainly restricted to smoking-related cancers, particularly lung cancer, raising concerns about residual confounding or effect modification by smoking.^25^^,^^26^ In the present study, we did not observe any associations between fruit intake and lung cancer mortality, in smokers or never smokers, in men or in women (of whom only 3% ever smoked). On the other hand, the association with digestive tract cancer mortality in the present study was much stronger than those reported in previous studies, especially those that assessed total rather than specifically fresh fruit consumption.^5^^,^^26^^,^^27^ The excess cancer mortality associated with fruit intake among a very small number of ex-regular alcohol drinkers in the present study is unexplained and might well be a chance finding or due chiefly to uncontrolled reverse causality.

In China, the incidence of COPD is high even among never smokers, due perhaps to poor nutrition and exposure to domestic biomass fuel use.^28^ Although there is suggestive evidence that consumption of antioxidants and antioxidant-rich food may slow the decline in lung function,^29^^,^^30^ prospective evidence on fruit intake and COPD risk is extremely limited. In a small European study of ∼ 3000 men with only 73 COPD deaths, each 100g daily portion of fruit was in a marginal association with a 14% (RR = 0.86, 95% CI 0.69–1.07) reduction in COPD mortality after adjustment for country, age, smoking, alcohol consumption, total energy intake and BMI.^31^ Our study included 15 times as many COPD deaths, and the association with fruit consumption was about twice as strong as in this European study.

The present study included a large number of deaths from several major vascular and non-vascular diseases, and the analyses have also dealt carefully with a range of confounding factors, reverse causality and regression dilution bias. The much stronger associations with fruit intake observed in our study, as compared with previous reports, may be accounted for by a number of other factors. First, we have focused only on fresh fruit, which is universally consumed raw in China, maximizing its potential health benefits.^16^^,^^32^ Second, the associations of fruit consumption with disease risks may be stronger in populations with low levels of consumption,^5^ and the average level of fruit consumption in our study population is much lower than that in most Western populations.^14^

We did not collect information on processed fruit, which is rarely consumed in China. About 95% of our participants reported daily consumption of fresh vegetables, and additionally adjusting for this and other dietary factors did not alter the results for fruit. The level of fruit consumption at baseline was assessed using a simple questionnaire, and the information on consumption amount was estimated indirectly through resurvey data. We were not able to check the agreement between self-reported and actual fruit consumption. However, the predictive validity^33^ of our fruit (not nutrient) consumption measure can be inferred from the observed strong associations of fruit intake with blood pressure and CVDs.^6^ Also the overall level and time trend of fruit consumption observed in our study were in line with the findings from national nutrition surveys based on three 24-h recalls (eTable 8, available as Supplementary data at *IJE* online).^3^ Furthermore, we did not collect information about total energy intake and types of fruit consumed, although the most commonly consumed fruits in China are apples, citrus fruits and pears. Our analyses have been adjusted for a range of potential confounders (including socioeconomic status, physical activity and BMI), but residual confounding may still exist. The somewhat stronger associations in rural women (eFigure 6), in whom few smoked or drank alcohol and most had similar socioeconomic status, however, may suggest that residual confounding should not play a major role.

There are several biologically plausible mechanisms supporting a causal relationship between higher fruit consumption and reduced mortality. Fruit is rich in vitamins, minerals, fibre, antioxidants and phytochemicals, which could help: reduce blood pressure (as observed in the present study) and platelet aggregation; improve glycaemic control, insulin sensitivity and lipid profile; stimulate the immune system and detoxification processes; and modulate hormone metabolism and systemic inflammation.^34^ It is also possible that fruit may optimize gut microbiota composition, leading to reduced risk of cardio-metabolic diseases.^35^ However, the exact underlying mechanisms can only be speculated upon and may well differ by disease.

In summary, the present study provides robust new evidence about the potential health benefits of regular fruit consumption on mortality from a range of major chronic diseases in Chinese adults. Despite the recent improvements, currently only less than 50% of the adult Chinese population consume fruit on a regular basis, which is much lower than in most Western populations. If the observed associations are largely causal and effective measures can be implemented to double the proportion of regular fruit consumers in China to the level seen in most Western populations, an estimated ∼ 530 000 (95% CI 344 000–676 000) deaths at ages 35–79 years and a potential of 270 000 deaths at age ≥ 80 years could be prevented each year (eTable 4), plus an even larger number of other non-fatal diseases that were not assessed directly in the present report. This suggests that improved dietary quality, especially increased fruit consumption, may represent an important and cost-effective strategy for preventing premature deaths in China.

## Supplementary Data

Supplementary data are available at *IJE* online.

## Supplementary Material

Supplementary Data
