# Supplementary material for: Fresh fruit consumption and all-cause and cause-specific mortality: findings from the China Kadoorie Biobank
Source: Int J Epidemiol. 2017 Apr 24;46(5):1444–55. doi: 10.1093/ije/dyx042 (PMC5837264; doi:10.1093/ije/dyx042)

**Online Materials**

**Table of contents**

Page 2. eTable 1. Calculation of the usual amount of fresh fruit consumption

Page 3. eTable 2. ICD-10 codes and distribution of deaths at 35-79 years in men and women

Page 4. eTable 3. Number of deaths and adjusted RRs for all-cause and cause-specific mortality by frequency of fresh fruit consumption

Page 5. eTable 4. Population attributable fraction (PAF) of low fruit consumption on all-cause and CVD mortality

Page6. eTable 5. Adjusted RRs (95% CI) for fatal and non-fatal COPD and site-specific cancer incidence contrasting regular to non-consumption and for 1 daily portion of fresh fruit, stratified by gender or alcohol consumption

Page 7. eTable 6. Adjusted RRs (95% CI) for major cause-specific mortality by fresh fruit consumption, using step-wise adjustment

Page 9. eTable 7. Adjusted RRs for major cause-specific mortality and COPD incidence by fresh fruit consumption, with different exclusions

Page 10. eTable 8. Distribution of self-reported fresh fruit consumption in the China Kadoorie Biobank (CKB) and China Health Nutrition Survey (CHNS)

Page 11. eFigure 1. Participant flow chart

Page 12. eFigure 2. Area-specific rate ratios (RRs) for all-cause mortality by fresh fruit consumption

Page 13. eFigure 3. Adjusted RRs for CVD mortality per 1 daily portion of fresh fruit consumption by baseline characteristics

Page 14. eFigure 4. Adjusted RRs for COPD mortality per 1 daily portion of fresh fruit consumption by baseline characteristics

Page 15. eFigure 5. Adjusted RRs for cancer mortality per 1 daily portion of fresh fruit consumption by baseline characteristics

Page 16. eFigure 6. Sex- & region-specific rate ratios (RRs) for all-cause mortality by fresh fruit consumption

**eTable 1. Calculation of the usual amount of fresh fruit consumption (based on 17,767 participants who attended the first resurvey in 2008)**

| Baseline  n | | 1^st^ resurvey (mean 2.6 years later)  **F** | | | | Consumption days per month | Mean daily portion* | Baseline consumption (portions/month)  **B** | Usual consumption (portions/month)†  **U** |
| --- | --- | --- | --- | --- | --- | --- | --- | --- | --- |
|  |  | Never/rarely  1 | Monthly  2 | Weekly  3 | Regularly  4 |  |  |  |  |
| 1 | Never/rarely | 16.72%  (194) | 58.10%  (674) | 17.07%  (198) | 8.10%  (94) | 0 | - | B_1_ = 0 | U_1_ = 7.17 |
| 2 | Monthly | 6.97%  (429) | 52.20%  (3213) | 29.39%  (1809) | 11.44%  (704) | 2.5 | 1.37 | B_2_ = 3.4  (2.5×1.37) | U_2_ = 9.76 |
| 3 | Weekly | 3.89%  (220) | 32.23%  (1821) | 37.59%  (2124) | 26.28%  (1485) | 8.6 | 1.43 | B_3_ = 12.3 (8.6×1.43) | U_3_ = 15.76 |
| 4 | Regularly | 1.25%  (60) | 11.62%  (558) | 24.57%  (1180) | 62.56%  (3004) | 23.6 | 1.62 | B_4_ = 38.2 (23.6×1.62) | U_4_ = 27.32 |

*The mean daily portion number came from the 2nd resurvey data, used as a proxy of baseline mean daily portion assuming that the daily portions of fresh fruit consumption did not vary much from the baseline to the 2^nd^ resurvey (i.e. people may change their frequency of fruit consumption but the daily amount of fresh fruit consumption remains roughly the same).

†Usual intake amount for each group was estimated by taking into account changes in consumption frequency between baseline and 1st resurvey using this formula Un = $\sum_{i=1}^{5} (Fni\times Bi)$; F is the percentage in each cell, B is the baseline proportion per month for each baseline category, U is the usual proportion per month for each baseline category.

**eTable 2: ICD-10 codes and distribution of deaths at 35-79 years in men and women**

| **Causes of death** | **ICD-10 codes** | **Men (n = 188,409)** | | |  | **Women (n = 273,933)** | | |  | **Total (n = 462,342)** | | |
| --- | --- | --- | --- | --- | --- | --- | --- | --- | --- | --- | --- | --- |
|  |  | **N** |  | **% death** |  | **N** |  | **% death** |  | **N** |  | **% death** |
| Ischemic heart disease | I20-I25 | 1175 |  | 11.1 |  | 863 |  | 11.8 |  | 2038 |  | 11.4 |
| Ischemic stroke | I63 | 354 |  | 3.4 |  | 231 |  | 3.2 |  | 585 |  | 3.3 |
| Intracerebral haemorrhage | I61 | 1302 |  | 12.3 |  | 1049 |  | 14.3 |  | 2351 |  | 13.1 |
| Other CVD | I00-I15, I28-I60, I62, I64-I88, I95-I99 | 655 |  | 6.2 |  | 537 |  | 7.3 |  | 1192 |  | 6.7 |
| **All CVD** | I00-I25, I28-I88, I95-I99 | 3486 |  | 33.0 |  | 2680 |  | 36.6 |  | 6166 |  | 34.5 |
|  |  |  |  |  |  |  |  |  |  |  |  |  |
| Digestive tract cancer | C15, C16, C18-C20 | 1528 |  | 14.5 |  | 737 |  | 10.1 |  | 2265 |  | 12.7 |
| Stomach cancer | C16 | 689 |  | 6.5 |  | 301 |  | 4.1 |  | 990 |  | 5.5 |
| Oesophageal cancer | C15 | 593 |  | 5.6 |  | 208 |  | 2.8 |  | 801 |  | 4.5 |
| Colorectal cancer | C18-C20 | 246 |  | 2.3 |  | 228 |  | 3.1 |  | 474 |  | 2.6 |
| Non-digestive tract cancer | C00-C14, C17, C21-C97 | 2584 |  | 24.5 |  | 1947 |  | 26.6 |  | 4531 |  | 25.3 |
| Lung cancer | C33-C34 | 1026 |  | 9.7 |  | 574 |  | 7.8 |  | 1600 |  | 8.9 |
| Liver cancer | C22 | 747 |  | 7.1 |  | 353 |  | 4.8 |  | 1100 |  | 6.1 |
| All other cancers | C00-C14, C17, C21, C23-C32, C35-C97 | 811 |  | 7.7 |  | 1020 |  | 13.9 |  | 1831 |  | 10.2 |
| **All cancer** | C00-C97 | 4112 |  | 38.9 |  | 2684 |  | 36.6 |  | 6796 |  | 38.0 |
|  |  |  |  |  |  |  |  |  |  |  |  |  |
| COPD | J41-J44, I26-I27 | 662 |  | 6.3 |  | 457 |  | 6.2 |  | 1119 |  | 6.3 |
| **All respiratory diseases** | J00-J99, I26-I27 | 829 |  | 7.8 |  | 543 |  | 7.4 |  | 1372 |  | 7.7 |
|  |  |  |  |  |  |  |  |  |  |  |  |  |
| **Other major chronic diseases** | D00-H95, K00-N99 | 701 |  | 6.6 |  | 524 |  | 7.2 |  | 1225 |  | 6.8 |
|  |  |  |  |  |  |  |  |  |  |  |  |  |
| **All other causes** | A00-B99, O00-Y99 | 1439 |  | 13.6 |  | 896 |  | 12.2 |  | 2335 |  | 13.1 |
| Transport accidents | V01-V99 | 464 |  | 4.4 |  | 260 |  | 3.5 |  | 724 |  | 4.0 |
|  |  |  |  |  |  |  |  |  |  |  |  |  |
| **Any** |  | 10,567 |  | 100 |  | 7327 |  | 100 |  | 17,894 |  | 100 |

**eTable 3: Number of deaths and adjusted RRs for all-cause and cause-specific mortality by frequency of fresh fruit consumption**

| **Causes of deaths** | **Never/rarely** | |  | **Monthly** | |  | **Weekly** | |  | **Regularly** | | ***P* for trend** |
| --- | --- | --- | --- | --- | --- | --- | --- | --- | --- | --- | --- | --- |
|  | **No. of deaths** | **RR (95% CI)** |  | **No. of deaths** | **RR (95% CI)** |  | **No. of deaths** | **RR (95% CI)** |  | **No. of deaths** | **RR (95% CI)** |  |
| IHD | 261 | 1.00 (0.88-1.14) |  | 878 | 0.86 (0.80-0.93) |  | 532 | 0.77 (0.71-0.84) |  | 367 | 0.63 (0.55-0.71) | <0.0001 |
| Ischemic stroke | 82 | 1.00 (0.79-1.26) |  | 255 | 0.80 (0.70-0.92) |  | 159 | 0.84 (0.72-0.99) |  | 89 | 0.67 (0.53-0.86) | 0.10 |
| Haemorrhagic stroke | 283 | 1.00 (0.88-1.13) |  | 1161 | 0.84 (0.79-0.90) |  | 640 | 0.77 (0.71-0.83) |  | 267 | 0.68 (0.59-0.77) | <0.0001 |
| Other CVD | 159 | 1.00 (0.85-1.18) |  | 484 | 0.79 (0.71-0.87) |  | 331 | 0.74 (0.66-0.82) |  | 218 | 0.66 (0.56-0.78) | 0.009 |
| **All CVD** | **785** | **1.00 (0.93-1.08)** |  | **2778** | **0.84 (0.80-0.87)** |  | **1662** | **0.77 (0.73-0.81)** |  | **941** | **0.66 (0.61-0.71)** | **<0.0001** |
|  |  |  |  |  |  |  |  |  |  |  |  |  |
| *Digestive tract cancer* | *308* | *1.00 (0.89-1.13)* |  | *955* | *0.82 (0.76-0.88)* |  | *597* | *0.74 (0.68-0.80)* |  | *405* | *0.72 (0.64-0.81)* | *0.004* |
| Oesophageal cancer | 154 | 1.00 (0.85-1.18) |  | 378 | 0.79 (0.71-0.88) |  | 192 | 0.79 (0.68-0.91) |  | 77 | 0.65 (0.50-0.83) | 0.03 |
| Stomach cancer | 113 | 1.00 (0.82-1.21) |  | 395 | 0.84 (0.75-0.94) |  | 282 | 0.79 (0.70-0.89) |  | 200 | 0.77 (0.66-0.91) | 0.17 |
| Colorectal cancer | 41 | 1.00 (0.73-1.38) |  | 182 | 0.88 (0.75-1.04) |  | 123 | 0.62 (0.52-0.73) |  | 128 | 0.69 (0.56-0.86) | 0.07 |
| *Non-digestive tract cancer* | *365* | *1.00 (0.90-1.11)* |  | *1686* | *0.96 (0.91-1.02)* |  | *1378* | *0.91 (0.86-0.96)* |  | *1102* | *0.91 (0.85-0.98)* | *0.19* |
| Lung cancer | 124 | 1.00 (0.83-1.20) |  | 593 | 1.05 (0.95-1.15) |  | 467 | 0.94 (0.86-1.02) |  | 416 | 1.02 (0.91-1.15) | 0.93 |
| Liver cancer | 97 | 1.00 (0.81-1.23) |  | 452 | 1.04 (0.94-1.16) |  | 322 | 0.95 (0.85-1.06) |  | 229 | 0.99 (0.85-1.15) | 0.64 |
| All other cancers | 144 | 1.00 (0.84-1.19) |  | 641 | 0.84 (0.77-0.92) |  | 589 | 0.85 (0.79-0.92) |  | 457 | 0.79 (0.70-0.88) | 0.12 |
| **All cancer** | **673** | **1.00 (0.92-1.08)** |  | **2641** | **0.90 (0.86-0.94)** |  | **1975** | **0.83 (0.80-0.87)** |  | **1507** | **0.83 (0.78-0.89)** | **0.007** |
|  |  |  |  |  |  |  |  |  |  |  |  |  |
| COPD | 191 | 1.00 (0.86-1.16) |  | 540 | 0.72 (0.66-0.79) |  | 275 | 0.61 (0.54-0.68) |  | 113 | 0.58 (0.47-0.71) | 0.0002 |
| **All respiratory diseases** | **214** | **1.00 (0.87-1.15)** |  | **620** | **0.74 (0.68-0.81)** |  | **342** | **0.62 (0.56-0.69)** |  | **196** | **0.68 (0.58-0.80)** | **0.006** |
|  |  |  |  |  |  |  |  |  |  |  |  |  |
| **Other major chronic diseases**^†^ | **140** | **1.00 (0.84-1.19)** |  | **505** | **0.85 (0.77-0.94)** |  | **383** | **0.80 (0.72-0.89)** |  | **197** | **0.67 (0.57-0.78)** | **0.002** |
|  |  |  |  |  |  |  |  |  |  |  |  |  |
| Transport accidents | 62 | 1.00 (0.77-1.30) |  | 285 | 0.83 (0.74-0.95) |  | 258 | 0.99 (0.88-1.12) |  | 119 | 0.93 (0.77-1.14) | 0.24 |
| **All other causes** | **242** | **1.00 (0.87-1.14)** |  | **969** | **0.85 (0.80-0.91)** |  | **744** | **0.86 (0.80-0.92)** |  | **380** | **0.76 (0.68-0.86)** | **0.03** |
|  |  |  |  |  |  |  |  |  |  |  |  |  |
| **All causes** | **2054** | **1.00 (0.96-1.05)** |  | **7513** | **0.85 (0.83-0.87)** |  | **5106** | **0.79 (0.76-0.81)** |  | **3221** | **0.73 (0.70-0.76)** | **<0.0001** |

CI: confidence interval; RR: mortality rate ratio;

Analyses were adjusted for age, sex, region, smoking, alcohol intake, education, income, consumption of meat, dairy products and preserved vegetables, survey season, physical activity and BMI.

† Includes deaths with known causes other than infectious diseases, CVD, cancer and respiratory diseases (see eTable 1 for detailed ICD-10 codes).

**eTable 4. Population attributable fraction (PAF) of low fruit consumption on all-cause and CVD mortality**

| **Mortality** | **PAF (%) calculation** | | | | |  | **Total deaths in China in 2013*** | | |  | **Deaths could be avoided through regular fresh fruit consumption for all population** | | |
| --- | --- | --- | --- | --- | --- | --- | --- | --- | --- | --- | --- | --- | --- |
|  | **RR (95% CI)** |  | **Pe** |  | **PAF** |  | **35-79 years** |  | **≥ 80 years** |  | **35-79 years** |  | **≥ 80 years**† |
| **All-cause** | 1.13 (1.08-1.18) |  | 0.82 |  | 9.4 |  | 5,631,637 |  | 2,834,884 |  | 529,374 |  | 266,479 |
| **CVD** | 1.24 (1.14-1.34) |  | 0.85 |  | 16.4 |  | 2,202,679 |  | 1,460,343 |  | 361,239 |  | 239,496 |

PAF is calculated as Pe x (1-1/RR), where Pe is the prevalence of non-regular fresh fruit consumption among those who died either from all-causes or due to CVD during follow-up.

* Data were kindly provided by Dr. Shiwei Liu from the China Global Burden of Disease Project Team.[^1^](#_ENREF_1)

† Estimated by applying the same RR and PAF at 35-79.

**Reference:**

**1.** Zhou M, Wang H, Zhu J, et al. Cause-specific mortality for 240 causes in China during 1990-2013: a systematic subnational analysis for the Global Burden of Disease Study 2013. *Lancet.* Oct 23 2015.

**eTable 5. Adjusted RRs (95% CI) for fatal and non-fatal COPD and site-specific cancer incidence contrasting regular to non-consumption and for 1 daily portion of fresh fruit, stratified by gender or alcohol consumption**

|  | **No. of cases** | **Regular vs. no- consumption** |  | **1 daily portion** |
| --- | --- | --- | --- | --- |
| **COPD** |  |  |  |  |
| Men | 4267 | 0.77 (0.70-0.84) |  | 0.72 (0.61-0.86) |
| Women | 4162 | 0.78 (0.71-0.85) |  | 0.69 (0.58-0.81) |
| **Total cancer** |  |  |  |  |
| Never drinkers | 11,463 | 0.91 (0.87-0.95) |  | 0.90 (0.82-0.99) |
| Ex-drinkers | 463 | 2.11 (1.72-2.59) |  | 1.79 (1.16-2.76) |
| Current drinkers | 2873 | 0.75 (0.69-0.83) |  | 0.77 (0.64-0.93) |
| **Digestive tract cancer** |  |  |  |  |
| Never drinkers | 3444 | 0.85 (0.78-0.93) |  | 0.87 (0.73-1.04) |
| Ex-drinkers | 154 | 1.69 (1.18-2.43) |  | 1.76 (0.84-3.70) |
| Current drinkers | 1148 | 0.72 (0.62-0.83) |  | 0.70 (0.52-0.93) |
| **Lung caner** |  |  |  |  |
| Never drinkers | 2037 | 0.96 (0.87-1.07) |  | 1.00 (0.80-1.26) |
| Ex-drinkers | 121 | 2.30 (1.49-3.54) |  | 1.42 (0.59-3.41) |
| Current drinkers | 666 | 0.86 (0.72-1.04) |  | 0.75 (0.52-1.10) |
| **Liver cancer** |  |  |  |  |
| Never drinkers | 1180 | 1.05 (0.91-1.22) |  | 0.99 (0.74-1.34) |
| Ex-drinkers | 97 | 1.64 (1.04-2.60) |  | 1.11 (0.42-2.89) |
| Current drinkers | 377 | 0.84 (0.65-1.09) |  | 0.89 (0.54-1.48) |
| **Other cancers** |  |  |  |  |
| Never drinkers | 5088 | 0.91 (0.85-0.97) |  | 0.88 (0.76-1.01) |
| Ex-drinkers | 111 | 4.11 (2.78-6.07) |  | 3.32 (1.37-8.04) |
| Current drinkers | 765 | 0.77 (0.65-0.91) |  | 0.97 (0.69-1.37) |

**eTable 6. Adjusted RRs (95% CI) for major cause-specific mortality by fresh fruit consumption, using step-wise adjustment**

| **Model** | **CVD** | | **Cancer** | | **COPD** | | **All-cause** | |
| --- | --- | --- | --- | --- | --- | --- | --- | --- |
|  | RR (95% CI)* | RR (95% CI)† | RR (95% CI)* | RR (95% CI)† | RR (95% CI)* | RR (95% CI)† | RR (95% CI)* | RR (95% CI)† |
| Age-,sex-, and region-adjusted | 0.53 (0.50-0.57) | 0.45 (0.39-0.51) | 0.70 (0.66-0.74) | 0.68 (0.61-0.77) | 0.36 (0.30-0.44) | 0.26 (0.18-0.37) | 0.58 (0.56-0.61) | 0.53 (0.49-0.57) |
| +Education and income | 0.61 (0.56-0.65) | 0.54 (0.47-0.62) | 0.77 (0.73-0.82) | 0.78 (0.69-0.88) | 0.46 (0.38-0.56) | 0.36 (0.25-0.52) | 0.67 (0.64-0.69) | 0.64 (0.59-0.69) |
| +Smoking and alcohol intake | 0.62 (0.58-0.67) | 0.55 (0.48-0.63) | 0.82 (0.77-0.87) | 0.83 (0.74-0.94) | 0.46 (0.38-0.56) | 0.36 (0.25-0.51) | 0.69 (0.66-0.72) | 0.66 (0.61-0.72) |
| +physical activity and BMI | 0.62 (0.57-0.67) | 0.54 (0.47-0.63) | 0.85 (0.80-0.90) | 0.86 (0.77-0.98) | 0.55 (0.45-0.67) | 0.46 (0.33-0.66) | 0.71 (0.68-0.74) | 0.68 (0.63-0.74) |
| **Full model*** | **0.66 (0.61-0.71)** | **0.61 (0.53-0.70)** | **0.83 (0.78-0.89)** | **0.84 (0.74-0.96)** | **0.58 (0.47-0.71)** | **0.51 (0.35-0.73)** | **0.73 (0.70-0.76)** | **0.72 (0.66-0.78)** |
| Model A1 | 0.66 (0.61-0.71) | 0.62 (0.53-0.71) | 0.84 (0.79-0.89) | 0.85 (0.75-0.96) | 0.59 (0.48-0.73) | 0.53 (0.37-0.76) | 0.74 (0.71-0.77) | 0.73 (0.67-0.79) |
| Model A2 | 0.66 (0.62-0.72) | 0.61 (0.53-0.71) | 0.84 (0.79-0.89) | 0.84 (0.74-0.96) | 0.58 (0.48-0.72) | 0.51 (0.35-0.73) | 0.74 (0.71-0.77) | 0.72 (0.67-0.78) |
| Model A3 | 0.66 (0.61-0.71) | 0.61 (0.53-0.70) | 0.83 (0.78-0.88) | 0.83 (0.74-0.95) | 0.59 (0.48-0.72) | 0.52 (0.36-0.75) | 0.73 (0.70-0.76) | 0.72 (0.66-0.78) |
| Model A4 | 0.66 (0.61-0.72) | 0.61 (0.53-0.71) | 0.84 (0.79-0.89) | 0.85 (0.75-0.96) | 0.58 (0.48-0.71) | 0.51 (0.35-0.73) | 0.74 (0.71-0.77) | 0.72 (0.67-0.78) |
| Model A5 | 0.66 (0.61-0.71) | 0.61 (0.53-0.70) | 0.83 (0.78-0.88) | 0.84 (0.74-0.95) | 0.59 (0.48-0.72) | 0.51 (0.35-0.73) | 0.73 (0.70-0.76) | 0.72 (0.66-0.78) |
| Model A6 | 0.67 (0.62-0.73) | 0.62 (0.54-0.72) | 0.83 (0.78-0.89) | 0.84 (0.74-0.95) | 0.61 (0.50-0.75) | 0.53 (0.37-0.77) | 0.74 (0.71-0.78) | 0.73 (0.67-0.79) |
| Model A7 | 0.67 (0.63-0.73) | 0.63 (0.55-0.73) | 0.84 (0.79-0.89) | 0.84 (0.75-0.96) | 0.59 (0.48-0.72) | 0.52 (0.37-0.75) | 0.75 (0.72-0.78) | 0.74 (0.68-0.80) |
| Model A8 | 0.68 (0.63-0.73) | 0.63 (0.55-0.73) | 0.83 (0.78-0.88) | 0.83 (0.74-0.94) | 0.59 (0.48-0.72) | 0.52 (0.36-0.74) | 0.74 (0.71-0.77) | 0.73 (0.67-0.79) |
| Model A9 | 0.66 (0.61-0.71) | 0.61 (0.53-0.70) | 0.86 (0.81-0.91) | 0.86 (0.76-0.97) | 0.60 (0.49-0.74) | 0.54 (0.37-0.77) | 0.74 (0.71-0.78) | 0.73 (0.67-0.79) |
| **Model** | **Other major chronic disease** | | **Digestive tract cancer** | | **Lung cancer** | | **Liver cancer** | |
|  | RR (95% CI)* | RR (95% CI)† | RR (95% CI)* | RR (95% CI)† | RR (95% CI)* | RR (95% CI)† | RR (95% CI)* | RR (95% CI)† |
| Age-,sex-, and region-adjusted | 0.49 (0.42-0.57) | 0.43 (0.32-0.58) | 0.60 (0.53-0.67) | 0.56 (0.45-0.69) | 0.76 (0.67-0.85) | 0.69 (0.54-0.88) | 0.85 (0.74-0.99) | 0.78 (0.58-1.04) |
| +Education and income | 0.57 (0.49-0.67) | 0.53 (0.39-0.72) | 0.66 (0.69-0.74) | 0.65 (0.52-0.81) | 0.85 (0.76-0.96) | 0.82 (0.64-1.04) | 0.93 (0.80-1.09) | 0.88 (0.65-1.19) |
| ++Smoking and alcohol intake | 0.58 (0.50-0.68) | 0.54 (0.40-0.73) | 0.70 (0.62-0.78) | 0.69 (0.56-0.86) | 0.97 (0.87-1.09) | 0.95 (0.75-1.21) | 0.96 (0.83-1.12) | 0.90 (0.67-1.22) |
| +++physical activity and BMI | 0.61 (0.52-0.71) | 0.56 (0.41-0.75) | 0.73 (0.65-0.82) | 0.73 (0.59-0.91) | 1.03 (0.92-1.16) | 1.02 (0.80-1.30) | 1.00 (0.86-1.16) | 0.94 (0.69-1.27) |
| **Full model*** | **0.67 (0.57-0.78)** | **0.62 (0.45-0.84)** | **0.72 (0.64-0.81)** | **0.72 (0.57-0.90)** | **1.02 (0.91-1.15)** | **0.99 (0.77-1.27)** | **0.99 (0.85-1.15)** | **0.93 (0.68-1.27)** |
| Model A1 | 0.68 (0.58-0.80) | 0.64 (0.47-0.88) | 0.73 (0.65-0.82) | 0.73 (0.58-0.91) | 1.02 (0.91-1.15) | 0.98 (0.76-1.26) | 0.99 (0.85-1.15) | 0.93 (0.68-1.26) |
| Model A2 | 0.67 (0.57-0.79) | 0.63 (0.46-0.86) | 0.73 (0.65-0.82) | 0.72 (0.58-0.90) | 1.01 (0.90-1.14) | 0.98 (0.76-1.25) | 0.99 (0.85-1.15) | 0.93 (0.68-1.27) |
| Model A3 | 0.67 (0.57-0.79) | 0.63 (0.46-0.86) | 0.72 (0.64-0.80) | 0.71 (0.56-0.89) | 1.02 (0.90-1.14) | 0.98 (0.76-1.25) | 0.99 (0.85-1.16) | 0.93 (0.68-1.27) |
| Model A4 | 0.67 (0.57-0.79) | 0.62 (0.46-0.85) | 0.73 (0.65-0.82) | 0.73 (0.58-0.91) | 1.03 (0.91-1.16) | 0.99 (0.77-1.27) | 1.00 (0.86-1.17) | 0.94 (0.69-1.28) |
| Model A5 | 0.68 (0.58-0.80) | 0.64 (0.47-0.87) | 0.72 (0.64-0.81) | 0.72 (0.57-0.90) | 1.00 (0.89-1.13) | 0.97 (0.76-1.25) | 0.98 (0.84-1.15) | 0.91 (0.67-1.24) |
| Model A6 | 0.70 (0.60-0.83) | 0.66 (0.49-0.91) | 0.71 (0.64-0.80) | 0.71 (0.57-0.89) | 1.02 (0.91-1.15) | 0.98 (0.76-1.25) | 1.00 (0.85-1.16) | 0.93 (0.68-1.27) |
| Model A7 | 0.72 (0.61-0.85) | 0.69 (0.51-0.94) | 0.72 (0.64-0.81) | 0.72 (0.57-0.90) | 1.03 (0.91-1.16) | 0.99 (0.77-1.27) | 0.98 (0.84-1.15) | 0.92 (0.67-1.25) |
| Model A8 | 0.67 (0.57-0.79) | 0.63 (0.46-0.86) | 0.71 (0.64-0.80) | 0.71 (0.56-0.89) | 1.02 (0.91-1.15) | 0.99 (0.77-1.27) | 0.97 (0.83-1.13) | 0.91 (0.66-1.24) |
| Model A9 | 0.69 (0.58-0.81) | 0.64 (0.47-0.88) | 0.75 (0.67-0.84) | 0.74 (0.59-0.93) | 1.01 (0.89-1.13) | 0.97 (0.76-1.25) | 1.02 (0.88-1.19) | 0.96 (0.70-1.31) |

*RR for regular fruit consumption vs. never/rarely fruit consumption; † RR for 1 extra daily portion of fresh fruit. Analyses were adjusted for age, sex and region, as strata variables, education and income, smoking and alcohol consumption, physical activity, BMI, survey season and consumption of meat, dairy products and preserved vegetables; Model A1-A9: On the basis of full model, additionally adjusted for fresh vegetables, tea, wheat, rice, other staple foods, poultry, fish, eggs (all in three categories) or soybean (4 categories), respectively.

**eTable 7. Adjusted RRs for major cause-specific mortality and COPD incidence by fresh fruit consumption, with different exclusions**

| **Causes of mortality** | **Never/rarely (n=27,534)** | |  | **Monthly (n=159,176)** | |  | **Weekly (n=147,155)** | | | | |  | **Regularly (n=128,477)** | | | |  | | **1 daily portion (n=462,342)** | | | | **P *_trend_*** |
| --- | --- | --- | --- | --- | --- | --- | --- | --- | --- | --- | --- | --- | --- | --- | --- | --- | --- | --- | --- | --- | --- | --- | --- |
|  | **No. of deaths** | **RR (95% CI)** |  | **No. of deaths** | **RR (95% CI)** |  | **No. of deaths** | | **RR (95% CI)** | | |  | **No. of deaths** | | **RR (95% CI)** | |  | | **No. of deaths** | | | **RR (95% CI)** |  |
| **Exclude first 2 years follow-up** | | | | | | | | | | | | | | | | | | | | | | | |
| All-cause | 1589 | 1.0 (0.95-1.05) |  | 5980 | 0.87 (0.84-0.89) |  | 4131 | | 0.81 (0.78-0.83) |  | | | 2645 | | 0.75 (0.72-0.78) |  | | | | **14,345** | **0.73 (0.67-0.80)** | | **<0.0001** |
| CVD | 620 | 1.0 (0.92-1.09) |  | 2265 | 0.86 (0.82-0.90) |  | 1358 | | 0.79 (0.75-0.83) |  | | | 783 | | 0.68 (0.62-0.74) |  | | | | **5026** | **0.62 (0.53-0.72)** | | **<0.0001** |
| COPD | 138 | 1.0 (0.84-1.20) |  | 421 | 0.75 (0.67-0.83) |  | 229 | | 0.67 (0.59-0.76) |  | | | 97 | | 0.66 (0.53-0.83) |  | | | | **885** | **0.63 (0.43-0.94)** | | **0.02** |
| Cancer | 531 | 1.0 (0.91-1.09) |  | 2093 | 0.89 (0.85-0.94) |  | 1616 | | 0.85 (0.81-0.89) |  | | | 1254 | | 0.84 (0.79-0.90) |  | | | | **5494** | **0.86 (0.75-0.99)** | | **0.03** |
| Digestive tract cancer | 243 | 1.0 (0.88-1.14) |  | 766 | 0.83 (0.77-0.90) |  | 493 | | 0.75 (0.68-0.82) |  | | | 332 | | 0.71 (0.62-0.80) |  | | | | **1834** | **0.69 (0.53-0.88)** | | **0.003** |
| Other major chronic disease | 104 | 1.0 (0.82-1.23) |  | 408 | 0.90 (0.81-1.00) |  | 311 | | 0.85 (0.76-0.95) |  | | | 164 | | 0.73 (0.61-0.87) |  | | | | **987** | **0.67 (0.48-0.94)** | | **0.02** |
| **Exclude those with prevalent disease at baseline*** | | | | | | | | | | | | | | | | | | | | | | | |
| All-cause | 1720 | 1.0 (0.95-1.05) |  | 6208 | 0.83 (0.81-0.85) |  | 4078 | 0.77 (0.75-0.80) | | |  | | | 2421 | 0.71 (0.68-0.74) |  | | | | **14,427** | **0.70 (0.64-0.76)** | | **<0.0001** |
| CVD | 692 | 1.0 (0.92-1.08) |  | 2368 | 0.81 (0.77-0.85) |  | 1375 | 0.75 (0.71-0.79) | | |  | | | 725 | 0.64 (0.59-0.70) |  | | | | **5160** | **0.60 (0.51-0.70)** | | **<0.0001** |
| COPD | 158 | 1.0 (0.84-1.18) |  | 426 | 0.68 (0.62-0.75) |  | 223 | 0.61 (0.53-0.70) | | |  | | | 90 | 0.64 (0.51-0.80) |  | | | | **897** | **0.62 (0.41-0.93)** | | **0.02** |
| Cancer | 556 | 1.0 (0.92-1.09) |  | 2160 | 0.88 (0.84-0.92) |  | 1551 | 0.82 (0.78-0.86) | | |  | | | 1128 | 0.80 (0.74-0.86) |  | | | | **5395** | **0.80 (0.69-0.92)** | | **0.002** |
| Digestive tract cancer | 251 | 1.0 (0.88-1.14) |  | 786 | 0.83 (0.77-0.89) |  | 479 | 0.76 (0.69-0.83) | | |  | | | 325 | 0.76 (0.67-0.87) |  | | | | **1841** | **0.77 (0.60-0.99)** | | **0.04** |
| Other major chronic disease | 109 | 1.0 (0.82-1.22) |  | 376 | 0.77 (0.69-0.87) |  | 278 | 0.75 (0.67-0.84) | | |  | | | 134 | 0.59 (0.49-0.72) |  | | | | **897** | **0.57 (0.40-0.83)** | | **0.003** |
| **Exclude those with self-rated poor health at baseline** | | | | | | | | | | | | | | | | | | | | | | | |
| All-cause | 1508 | 1.0 (0.95-1.05) |  | 6171 | 0.86 (0.84-0.89) |  | 4331 | 0.81 (0.79-0.83) | | |  | | | 2849 | 0.77 (0.73-0.80) |  | | | | **14,859** | **0.76 (0.70-0.83)** | | **<0.0001** |
| CVD | 566 | 1.0 (0.92-1.09) |  | 2203 | 0.84 (0.80-0.88) |  | 1378 | 0.78 (0.74-0.82) | | |  | | | 820 | 0.69 (0.63-0.75) |  | | | | **4967** | **0.65 (0.56-0.77)** | | **<0.0001** |
| COPD | 112 | 1.0 (0.82-1.22) |  | 373 | 0.72 (0.64-0.80) |  | 217 | 0.68 (0.60-0.78) | | |  | | | 92 | 0.65 (0.52-0.82) |  | | | | **794** | **0.68 (0.45-1.03)** | | **0.07** |
| Cancer | 541 | 1.0 (0.92-1.09) |  | 2308 | 0.90 (0.86-0.94) |  | 1725 | 0.82 (0.79-0.86) | | |  | | | 1358 | 0.83 (0.78-0.89) |  | | | | **5932** | **0.84 (0.73-0.96)** | | **0.01** |
| Digestive tract cancer | 246 | 1.0 (0.88-1.14) |  | 834 | 0.82 (0.76-0.89) |  | 521 | 0.73 (0.67-0.79) | | |  | | | 370 | 0.72 (0.64-0.82) |  | | | | **1971** | **0.72 (0.56-0.91)** | | **0.01** |
| Other major chronic disease | 93 | 1.0 (0.81-1.24) |  | 388 | 0.88 (0.78-0.98) |  | 302 | 0.87 (0.78-0.97) | | |  | | | 160 | 0.73 (0.61-0.88) |  | | | | **943** | **0.70 (0.50-0.99)** | | **0.04** |
| **Exclude first 2 years follow-up and those with self-rated poor health or prevalent disease at baseline** | | | | | | | | | | | | | | | | | | | | | | | |
| All-cause | 1042 | 1.0 (0.94-1.07) |  | 4249 | 0.87 (0.84-0.90) |  | 2958 | 0.83 (0.80-0.86) | | |  | | | 1810 | 0.76 (0.71-0.80) |  | | **10,059** | | | **0.75 (0.67-0.83)** | | **<0.0001** |
| CVD | 415 | 1.0 (0.90-1.11) |  | 1563 | 0.82 (0.77-0.87) |  | 965 | 0.77 (0.72-0.82) | | |  | | | 536 | 0.67 (0.61-0.74) |  | | **3479** | | | **0.65 (0.54-0.78)** | | **<0.0001** |
| COPD | 71 | 1.0 (0.78-1.28) |  | 253 | 0.74 (0.65-0.85) |  | 159 | 0.80 (0.68-0.93) | | |  | | | 64 | 0.83 (0.63-1.09) |  | | **547** | | | **1.01 (0.62-1.64)** | | **0.96** |
| Cancer | 373 | 1.0 (0.90-1.11) |  | 1577 | 0.89 (0.84-0.95) |  | 1177 | 0.84 (0.79-0.89) | | |  | | | 872 | 0.81 (0.75-0.88) |  | | **3999** | | | **0.82 (0.69-0.96)** | | **0.01** |
| Digestive tract cancer | 170 | 1.0 (0.85-1.17) |  | 584 | 0.85 (0.78-0.93) |  | 361 | 0.76 (0.68-0.84) | | |  | | | 251 | 0.74 (0.64-0.86) |  | | **1366** | | | **0.72 (0.54-0.97)** | | **0.03** |
| Other major chronic disease | 57 | 1.0 (0.76-1.32) |  | 265 | 0.93 (0.81-1.07) |  | 198 | 0.91 (0.79-1.04) | | |  | | | 99 | 0.73 (0.58-0.92) |  | | **619** | | | **0.66 (0.42-1.02)** | | **0.06** |
|  |  |  |  |  |  |  |  |  | | |  | | |  |  |  | |  | | |  | |  |
| **COPD incidence** |  |  |  |  |  |  |  |  | | |  | | |  |  |  | |  | | |  | |  |
| Main analysis | 714 | 1.0 (0.93-1.08) |  | 3706 | 0.93 (0.90-0.97) |  | 2722 | 0.86 (0.83-0.89) | | |  | | | 1287 | 0.77 (0.72-0.82) |  | | **8429** | | | **0.70 (0.63-0.79)** | | **<0.0001** |
| Sensitivity analysis† | 418 | 1.0 (0.90-1.11) |  | 2606 | 0.93 (0.89-0.97) |  | 1882 | 0.86 (0.83-0.90) | | |  | | | 808 | 0.74 (0.68-0.80) |  | | **5714** | | | **0.66 (0.57-0.76)** | | **<0.0001** |

* Including those with a history of self-reported physician-diagnosed rheumatic heart disease, rheumatoid arthritis, tuberculosis, asthma, cirrhosis, chronic hepatitis, peptic ulcer, gall/gall bladder stone, kidney disease, psychiatric disorders, or neurasthenia. † Exclude first 2 years follow-up and those with self-rated poor health or prevalent disease at baseline.

**eTable 8. Distribution of self-reported fresh fruit consumption in the China Kadoorie Biobank (CKB) and China Health Nutrition Survey (CHNS)**

|  | **CKB** | | | | | | | | | |
| --- | --- | --- | --- | --- | --- | --- | --- | --- | --- | --- |
|  | Baseline (2004-08)  N= 512,891 | | | 1^st^ resurvey (2008)  N=19,788 | | | | 2^nd^ resurvey (2013-14)  N=25,069 | | |
| % of regular fresh fruit consumption | 28.2 | | | 30.4 | | | | 45.1 | | |
|  | **CHNS*** | | | | | | | | | |
| Year of survey | 1991 | 1993 | 1997 | | 2000 | 2004 | 2006 | | 2009 | 2011 |
| Consumption rate (%) | 11.2 | 9.8 | 11.4 | | 12.7 | 18.5 | 24.8 | | 33.4 | 47.2 |
| Consumption amount (g/d) | 10.2 | 12.7 | 20.7 | | 16.8 | 24.9 | 57.7 | | 57.7 | 86.4 |

*Only results for 30-44 years of age group are presented here (i.e. not those for 18-29 years); consumption was collected using 3 consecutive 24-hour recalls. Xiao et al. Trends of vegetables and fruits consumption among Chinese adults aged 18-44 years old from 1991 to 2011. Chin J Epidemiol, March 2015, Vol.36, No.3 Page 232-236.

**eFigure 1. Participant flow chart**

Excluded 13,289 participants with prior history of chronic obstructive respiratory disease*

Excluded 16,162 participants with prior history of diabetes*

Excluded 2577 participants with prior history of cancer*

Excluded 8884 participants with prior history of stroke*

Excluded 15,472 participants with prior history of ischemic heart disease*

462,342 participants included in the China Kadoorie Biobank study

12,668 volunteers aged 30 - 34 years or 75 - 79 years were also included.

512,891 participants included in the China Kadoorie Biobank study

500,223 participants participated

1,801,167 participants, aged 35-74 years, were invited

**eFigure 2. Area-specific rate ratios (RRs) for all-cause mortality by fresh fruit consumption**


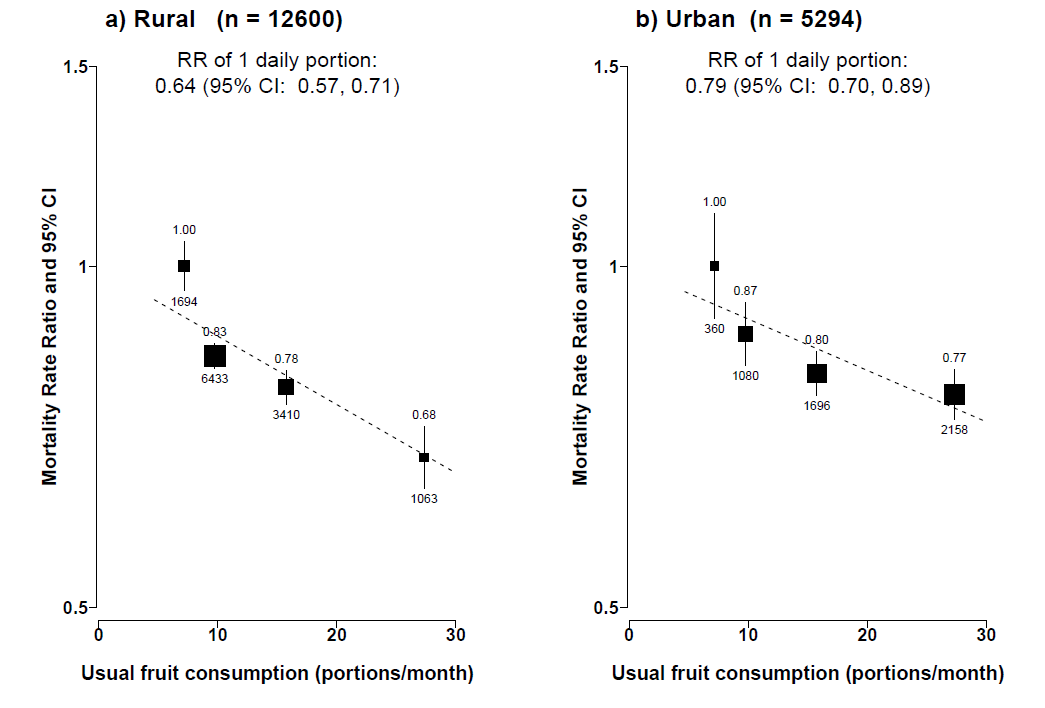


**eFigure 3. Adjusted RRs for CVD mortality per 1 daily portion of fresh fruit consumption by baseline characteristics**


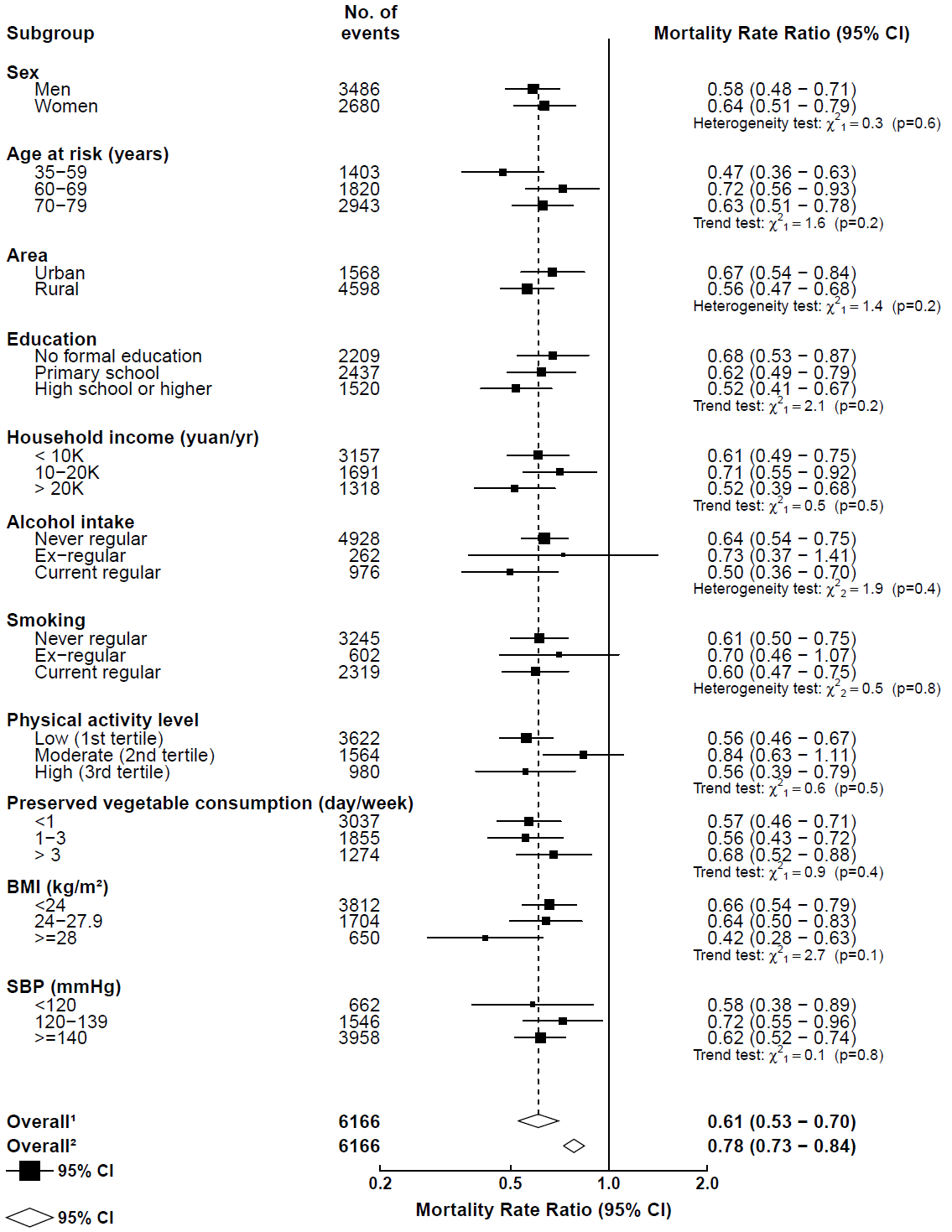


**eFigure 4. Adjusted RRs for COPD mortality per 1 daily portion of fresh fruit consumption by baseline characteristics**


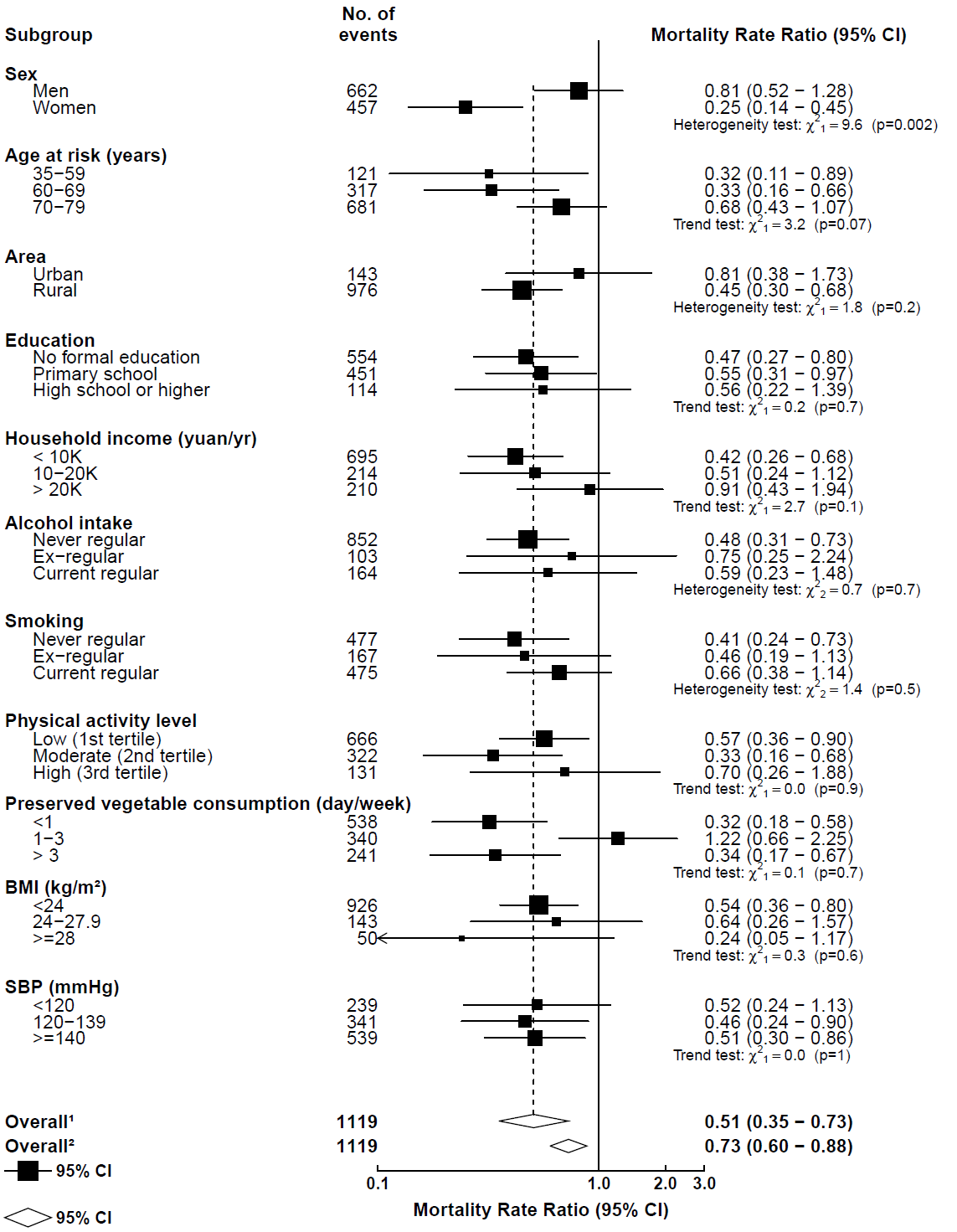


**eFigure 5. Adjusted RRs for cancer mortality per 1 daily portion of fresh fruit consumption by baseline characteristics**


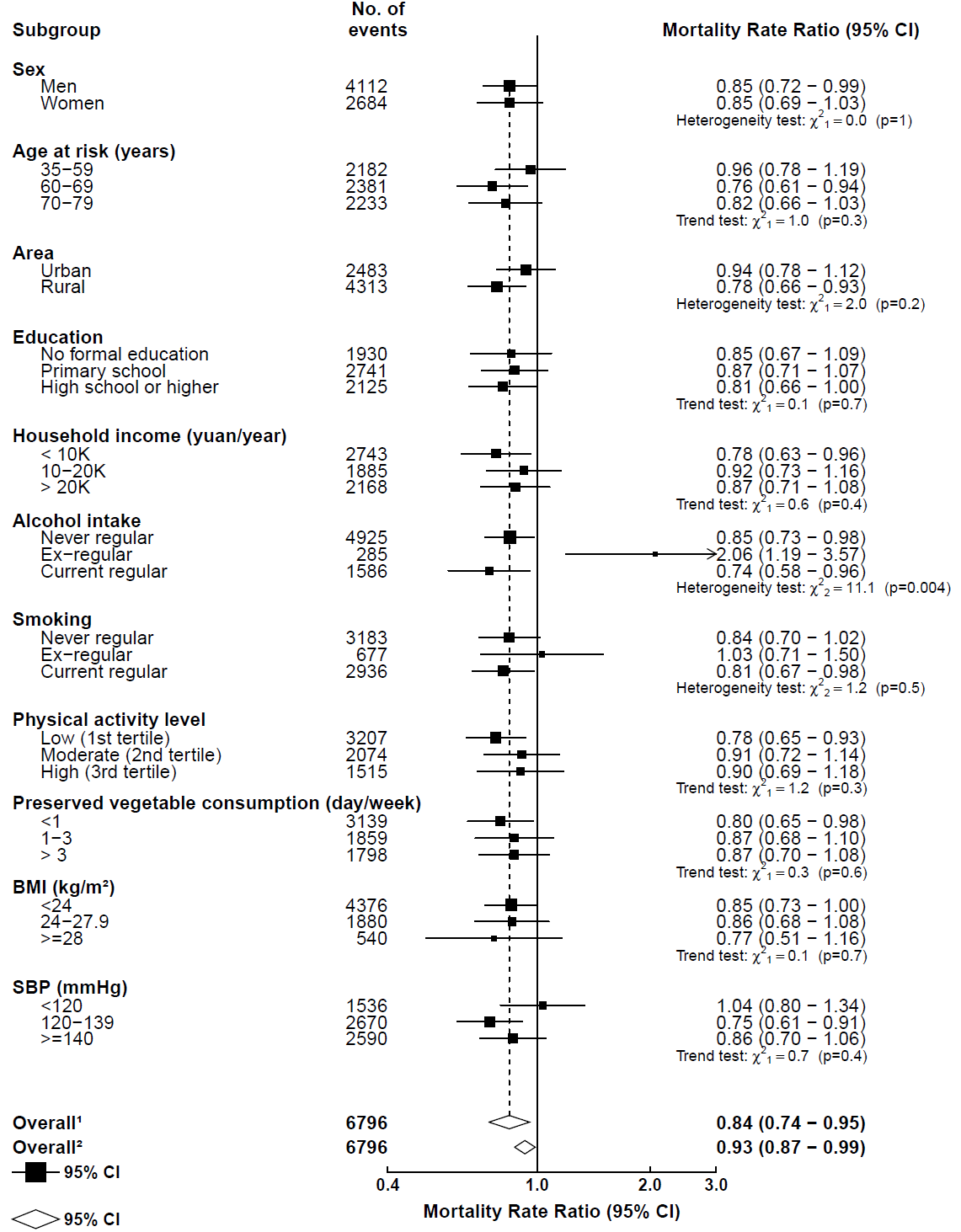


**eFigure 6. Sex- & region-specific rate ratios (RRs) for all-cause mortality by fresh fruit consumption**


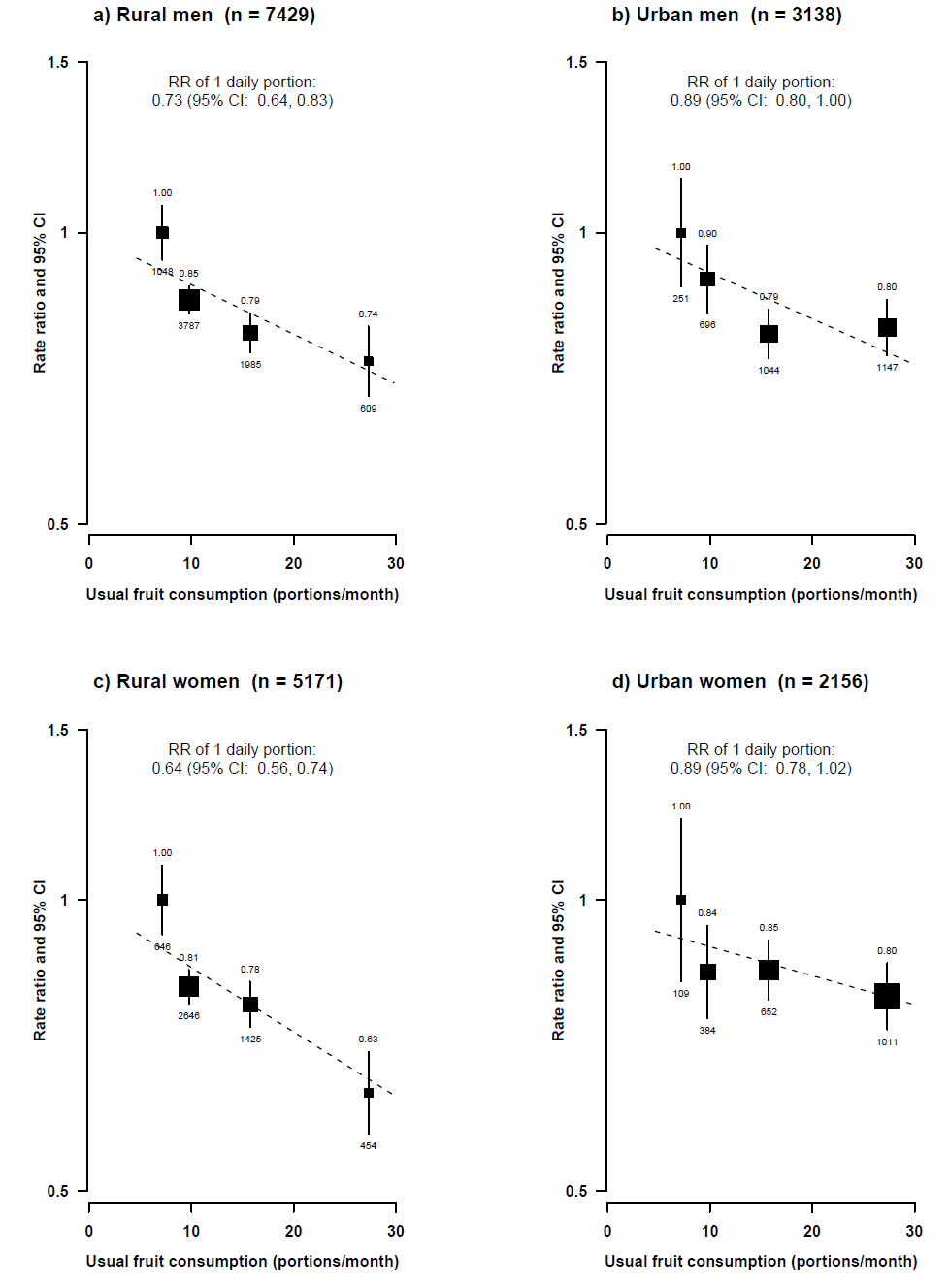

Supplement: Supplementary Data [file dyx042_ije-2016-08-0946-file006.docx]
